# Calcium currents in striatal fast-spiking interneurons: dopaminergic modulation of Ca_V_1 channels

**DOI:** 10.1186/s12868-018-0441-0

**Published:** 2018-07-16

**Authors:** Ernesto Alberto Rendón-Ochoa, Teresa Hernández-Flores, Victor Hugo Avilés-Rosas, Verónica Alejandra Cáceres-Chávez, Mariana Duhne, Antonio Laville, Dagoberto Tapia, Elvira Galarraga, José Bargas

**Affiliations:** División de Neurociencias, Instituto de Fisiología Celular, Universidad Nacional Autónoma de México, Circuito Exterior s/n Ciudad Universitaria, Col. Coyoacán, 04510 Ciudad de México, México

**Keywords:** Ca^2+^-currents, Ca^2+^-channels, Fast-spiking interneurons, Dopamine, D1-like dopamine receptors, Excitability

## Abstract

**Background:**

Striatal fast-spiking interneurons (FSI) are a subset of GABAergic cells that express calcium-binding protein parvalbumin (PV). They provide feed-forward inhibition to striatal projection neurons (SPNs), receive cortical, thalamic and dopaminergic inputs and are coupled together by electrical and chemical synapses, being important components of the striatal circuitry. It is known that dopamine (DA) depolarizes FSI via D_1_-class DA receptors, but no studies about the ionic mechanism of this action have been reported. Here we ask about the ion channels that are the effectors of DA actions. This work studies their Ca^2+^ currents.

**Results:**

Whole-cell recordings in acutely dissociated and identified FSI from PV-Cre transgenic mice were used to show that FSI express an array of voltage gated Ca^2+^ channel classes: Ca_V_1, Ca_V_2.1, Ca_V_2.2, Ca_V_2.3 and Ca_V_3. However, Ca_V_1 Ca^2+^ channel carries most of the whole-cell Ca^2+^ current in FSI. Activation of D_1_-like class of DA receptors by the D_1_-receptor selective agonist SKF-81297 (SKF) enhances whole-cell Ca^2+^ currents through Ca_V_1 channels modulation. A previous block of Ca_V_1 channels with nicardipine occludes the action of the DA-agonist, suggesting that no other Ca^2+^ channel is modulated by D_1_-receptor activation. Bath application of SKF in brain slices increases the firing rate and activity of FSI as measured with both whole-cell and Ca^2+^ imaging recordings. These actions are reduced by nicardipine.

**Conclusions:**

The present work discloses one final effector of DA modulation in FSI. We conclude that the facilitatory action of DA in FSI is in part due to Ca_V_1 Ca^2+^ channels positive modulation.

## Background

Inhibitory GABAergic interneurons are part of striatal circuitry. They control striatal projection neurons output (SPNs), are a part of neuronal ensembles and participate in cognition, procedural learning and motor performance [1–8]. Among all striatal interneurons, parvalbumin-positive (PV+) fast spiking interneurons (FSI) are the most studied. They can fire at high frequencies with little adaptation and represent about 0.7% of the total neuronal population. Although the proportion of PV+ interneurons is small compared to spiny projection neurons (SPN), they have physiological relevance by providing feed forward perisomatic and dendritic inhibition to large numbers of SPNs [1, 2, 9]. FSI receive inputs from cortical and thalamic regions [3, 10, 11], are interconnected by gap junctions and GABAergic chemical synapses that may help to generate synchronized or correlated firing between them. Activation of FSI has wide-spread effects upon SPNs [12, 13].

Striatal neurons receive massive dopaminergic innervation from the substantia nigra pars compacta (SNc) [14–16]. In vitro studies have shown that dopamine is an important modulator in the striatum which shapes excitability and circuitry management through, in part, the control of different receptors, ion channels, such as K^+^, Ca^2+^ and synaptic channels, neurons and neuronal ensembles [17–19]. In FSI, DA binds to D5-type dopamine receptors, a member of the D1-class receptors [20, 21]. Activation of these receptors produces a depolarization accompanied by action-potential (AP) discharge in striatal FSI [20, 21], as well as in FSI from the prefrontal cortex [22, 23] and basolateral amygdala [24]. Although DA receptors expressed in striatal FSI are known, no description about their functional effectors has been made. In SPNs, dopamine modulates Ca^2+^ entry through somatic Ca_V_1, Ca_V_2.1 and Ca_V_2.2 currents [25, 26] regulating firing frequency [25, 27]. In striatal cholinergic interneurons (CHI), dopamine modulates whole-cell Ca^2+^ current regulating firing properties, as well as the time course and shape of action potentials (AP) [28]. However, no study has been made to know whether calcium channels are involved on the depolarization produced by dopamine in FSI. Hence, this study was propose to find out: (1) the Ca^2+^ channel classes expressed in FSI, (2) if there is dopaminergic modulation of Ca^2+^ currents in FSI, and finally, (3) whether there are particular Ca^2+^ channels modulated by dopamine receptors. Accordingly, as a first approach, we use whole-cell recording in acutely dissociated striatal and identified FSI obtained from transgenic PV-cre mice in order to avoid indirect actions. Besides, whole cell current clamp recordings in slices as well as dynamic Ca^2+^ imaging with single cell resolution were performed. All techniques confirmed the hypothesis that D1-class receptor agonists enhance Ca^2+^ current carried by Ca_V_1 channel leading to an increase in excitability of striatal FSI.

## Methods

### Experimental subjects and design

**Fig. 1:**
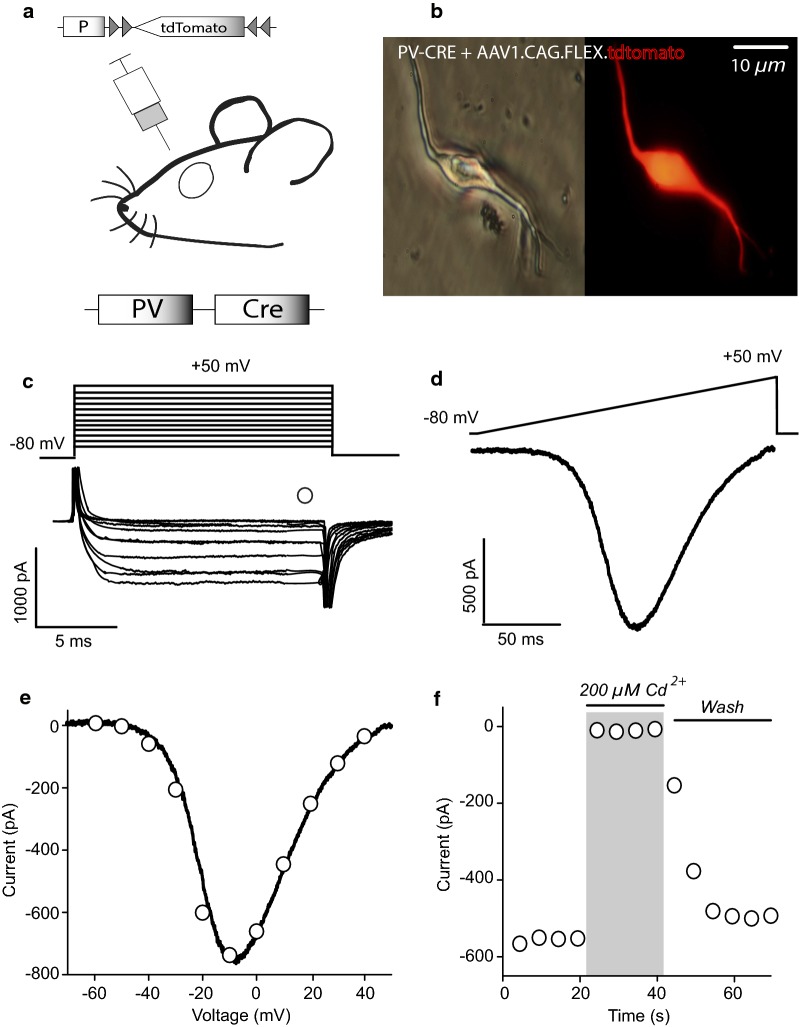
Whole-cell Ca^2+^ currents in acutely dissociated FSI. **a** Schematic infection protocol in PV-cre mice with a viral construction containing tdTomato into the dorsal striatum. **b** Representative images of virally infected, acutely dissociated PV-cre FSI. Left: light microscopy; right: the same fluorescent tdTomato PV-cre cell. **c** Inward currents (bottom) elicited by rectangular voltage commands from − 80 to 50 mV (top) in 10 mV steps (tail currents are clipped). Empty circle shows where the amplitude current measurements were obtained. **d** Inward current in the same neuron elicited by a ramp command from − 80 to 50 mV (0.7 mV/ms). **e** Current–voltage relationship (I–V plot). Empty circles are measurements taken from currents elicited with voltage commands (as in **c**) and continuous line was the current obtained with the ramp command (as in **d**). Measurements using both protocols are superimposed. Note that measurements using the ramp command appear to “fit” measurements using the square commands suggesting good voltage control and space clamp. **f**  Representative time course of Ca^2+^ current blockade during bath application of 200 µM Cd^2+^

**Fig. 2:**
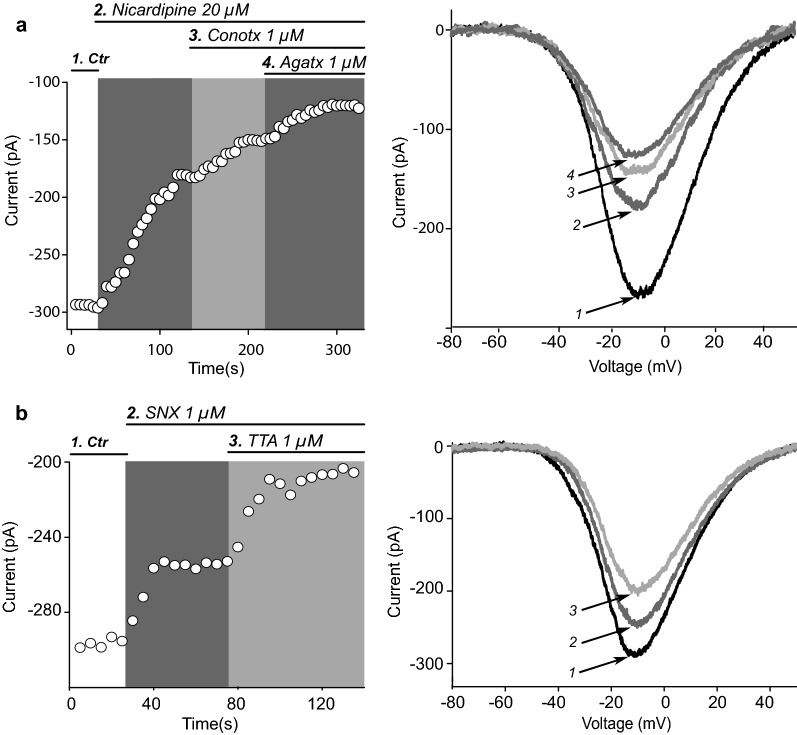
Calcium channels expressed in striatal FSI. **a** Left: representative time course of peak maximum Ca^2+^ current amplitude during the sequential addition of 20 µM nicardipine, a Ca_V_1 (L) channel antagonist, 1 µM ω-conotoxin GIVA (ω-CgTx), a Ca_V_2.2 (N) channel antagonist, and 1 µM ω-agatoxin TK (ω-AgTx), a Ca_V_2.1 (P/Q) channel antagonist. Right: Representative I–V plots obtained during sequential application of each Ca^2+^ channel antagonist and the consequent Ca^2+^ current reduction. Note remaining unblocked current. **b** Left: Time course of maximum Ca^2+^ current amplitude showing the action of saturating concentrations of 1 µM SNX-482 (SNX), a Ca_V_2.3 (R) channel antagonist and 1 µM TTA-P2 (TTA), a Ca_V_3 (T) channel antagonist. Right: Representative I–V plots during sequential application of Ca^2+^ channel antagonists with the consequent Ca^2+^ current reduction. Average percentage of current reduction in a sample of experiments after each channel antagonist was taken as the contribution of a specific Ca^2+^ channel class as seen in Table 1

**Fig. 3:**
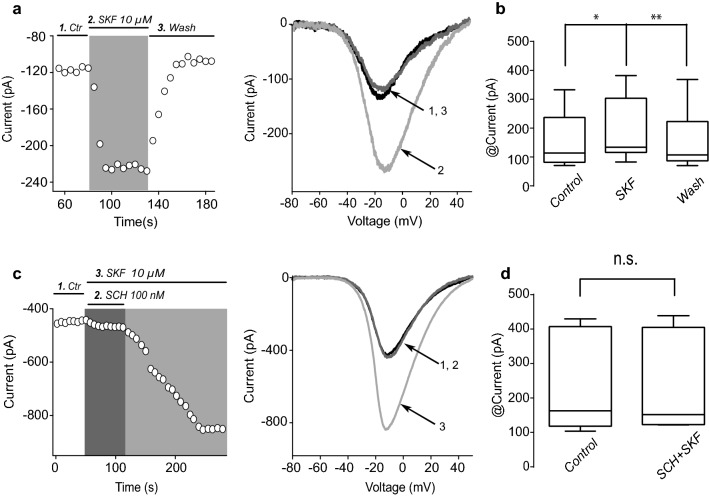
Activation of D1-class DA receptors enhances whole-cell Ca^2+^ currents in FSI. **a** Left: Representative time course (left) and representative I–V plots (right) showing that activation of D1-like DA receptors by addition of the selective DA agonist 10 µM SKF-81297 (SKF) to the bath solution enhances control Ca^2+^ currents. **b** Box plots summary of absolute Ca^2+^ current amplitudes in control, during SKF and after washing the agonist (*n* = 8; Friedman ANOVA test *F*_2,14_ = 13, P = 0.0003; *P < 0.05, **P < 0.01; post hoc Dunn’s multiple comparisons test). **c** Time course of maximum Ca^2+^ current showing specific blockade of SKF actions by the selective DA receptor antagonist 100 nM SCH-23390 (SCH) in the presence of SKF. Removal of SCH leads to an enhancement of Ca^2+^ current by SKF. Representative I–V plots at right. **d** Box plot summarizing the absolute current amplitude in control conditions and during addition of SCH plus SKF (n = 8; P = 0.99; Wilcoxon T test)

**Fig. 4:**
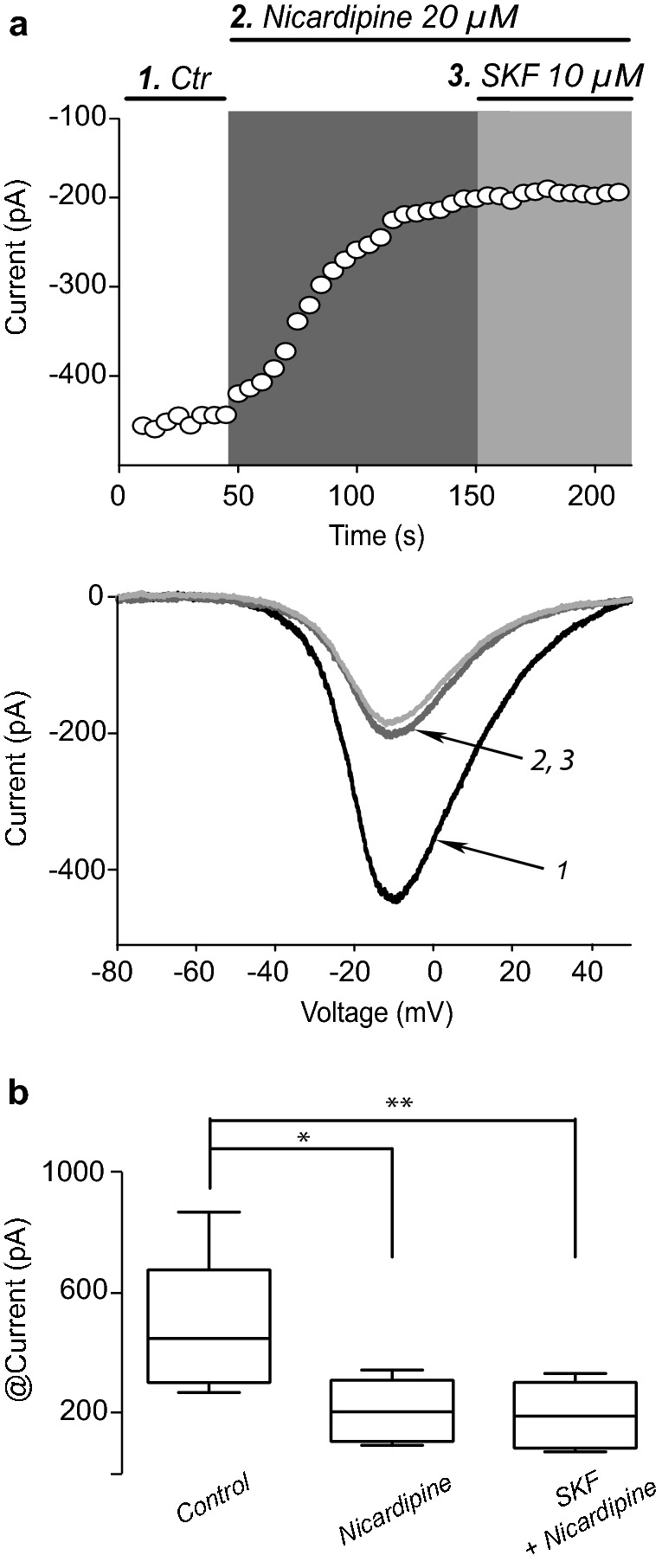
Dopamine D_1_-class agonist acts via Ca_V_1 Ca^2+^ currents modulation. **a** Representative time course (top) and a representative I–V plots (bottom) showing that addition of SKF fails to enhance whole-cell Ca^2+^ current when Ca_V_1 channels are previously blocked by the selective antagonist nicardipine (20 µM), suggesting that dopaminergic modulation mainly facilitates Ca_V_1 Ca^2+^ currents. **b** Box plots of a sample of similar experiments (*n* = 8. Friedman ANOVA test *F*_2,14_ = 13, P = 0.0003; *P< 0.05, **P < 0.01; post hoc Dunn’s multiple comparisons test)

**Fig. 5:**
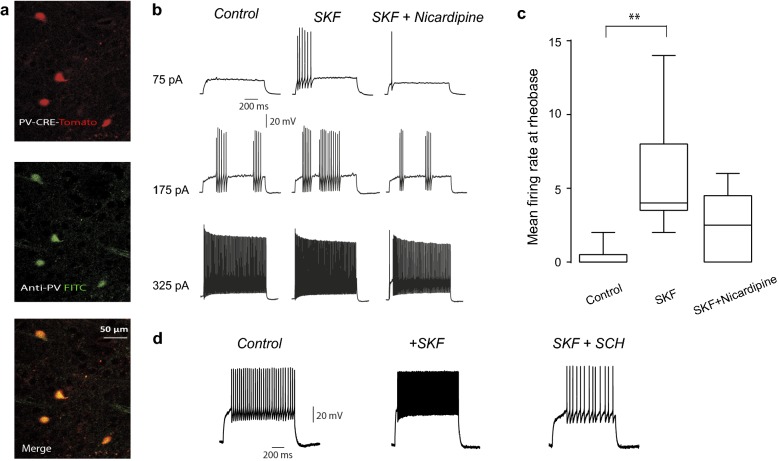
Excitability increase by SKF in current clamp experiments is occluded by nicardipine. **a** Top: immunocytochemical preparation showing striatal fluorescent neurons from a PV-cre mouse infected with adeno-associated virus with tdTomato (red). Middle: Corroboration by a fluorescein isothiocyanate (FITC) conjugated antibody against PV (green). Bottom: Merge. **b** Evoked firing to different stimulus strengths (somatic current injections values at left). Note that bath application of 10 μM SKF increased firing rate and this action is reversed by 20 μM nicardipine, suggesting that increases in firing are due to Ca_V_1 channels. **c** Summary of changes in a sample of neurons in which mean firing rate at rheobase was compared (n = 6; P < 0.0021; Friedman ANOVA with post hoc Dunn’s test using average firing rate at rheobase). **d** Bath application of SKF increased mean firing rate an effect which was reversed by 100 nM SCH (n = 4)

**Fig. 6:**
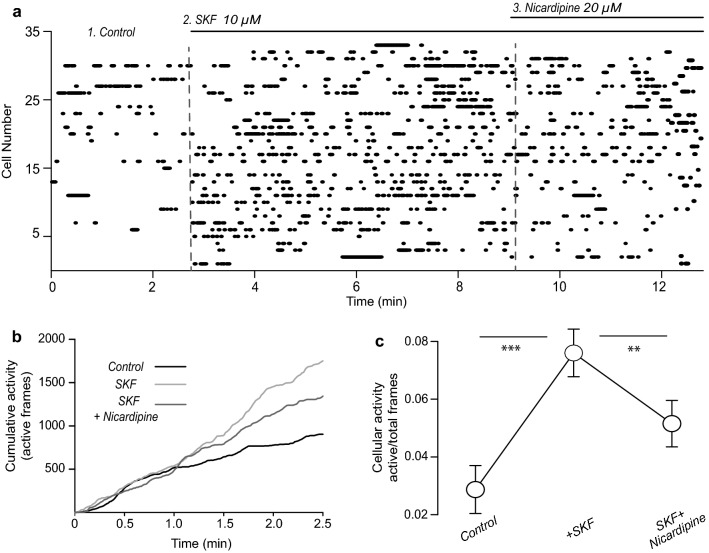
SKF increases activity of FSI-PV+ in the striatal microcircuit as seen with Ca^2+^ imaging. **a** Raster plot of several FSI activity (n = 33 FSI identified from PV-cre mice obtained from 6 different experiments/slices from 3 different mice). Fluorescense induced by Ca^2+^ entry allows infer electrical activity (see Perez-Ortega et al. [6]). Dots in each row of the raster show the activity of a single FSI, at different epochs during the experiment, separated by dashed vertical lines. Left panel: FSI activity in control conditions. Middle panel: addition of 10 µM SKF increases the number of FSI exhibiting spontaneous activity. Note that previous to SKF administration several FSI were silent. Right panel: addition of nicardipine in the continuous presence of SKF reduces the number of active FSI neurons. The experiment demonstrates that DA D1-like receptor activation enhances the number active FSI neurons within the striatal microcircuit in part by facilitating Ca_V_1 Ca^2+^ currents. **b** Summary of cumulative activity from A. **c** Summary of activity probability in each condition. Note that nicardipine does not completely reverse SKF actions (Friedman ANOVA; *F*_2, 64_ = 29.63, P < 0.0001; **P < 0.01, ***P < 0.001; with post hoc Dunn’s tests)

Experimental subjects, obtained from IFC bioterium were: B6; 12P2-*Pvalb*^*tm*1(*cre*)*Arbr*^/J (PV-Cre; Silvia Arber, Friederich Miescher Institute; Jackson Labs, stock# 008069), called PV+ mice from now on. Experimental subjects were housed in acrilic cages (4–5 mice per cage; 19 × 29 × 12 cm) with wood-based bedding and cardboard cylinders, kept on a 12:12 light/dark (light beginning at 8 am) period with a temperature maintained at 20–21 °C in IFC vivarium after surgery (see below) until used for experiments. All animals had standard rodent chow and water ad libitum. In order to identify isolated PV+ interneurons, PV-Cre transgenic mice at PD 21 (21 days, mean ± 4 days, 30 g mean ± 4, at 14–18 h), were anesthetized i.p. with ketamine (Bayer 75 mg/kg) and xilazine (Bayer 10 mg/kg) and injected stereotaxically in a laminar flow hood (Telsar technologies. Model PV-30/60) in a dedicated, sterile room, with the following viral constructs (University of Pennsylvania Vector Core): AAV2/1.CAG.Flex.tdTomato.WPRE.bGH (Honguki Zeng) for whole cell recordings in isolated cells, AAV1.Syn.Flex.GCaMP6f.WPRE.SV40 [29], for calcium imaging recordings and AAV1.CAG.Flex.eGFP.WPRE.bGH (Allen institute) for some current clamp experiments in slices at the following coordinates relative to bregma (in mm): AP = 0.9, ML = ± 1.2, DV = − 3.2. The total virus volume injected was 0.8 µl over a period of 10 min (Fig. 1a). Animals were monitored for two weeks to ensure full recovery and fluorescent protein expression (Fig. 1b). A total of 45 infected PV-Cre mice were randomly assigned to 6 independent groups: for voltage clamp recordings of calcium currents (see next sections for details of the techniques) to observe contribution of Ca^2+^ channels classes (Fig. 2; n = 19 recordings from 18 different mice, below); effects of DA on Ca^2+^ currents (Fig. 3a, b; n = 8 recording from 8 different mice); SCH + SKF control group (Fig. 3c, d; n = 6 recordings from 4 different mice); nicardipine on DAergic actions (Fig. 4; n = 8 recordings from 6 different mice); current clamp recordings in slices (Fig. 5; n = 6 recordings from 6 different mice for SKF-nicardipine experiments and n = 4 for SKF-SCH experiments) and calcium imaging experiments (Fig. 6; n = 33; for imaging PV-cre identified FSI were extracted from 6 different experiments/slices from 3 different mice). The experimental units were single neuron recordings or changes in fluorescence (∆F/F where ∆F = changes in fluorescence and F = basal fluorescence). Subject numbers were minimized to obtain statistical significance.

### Preparation of dissociated neurons and slices

Brain slices and acutely dissociated neurons were obtained and described in previous work [30–34]. Briefly, infected PV-Cre mice were anesthetized (see above). The mice were decapitated, their brains were removed and submerged in iced saline solution containing (in mM): 126 NaCl, 3 KCl, 26 NaHCO_3_, 2 CaCl_2_, 1 MgCl_2_, 11 glucose, 0.2 thiourea and 0.2 of ascorbic acid (25 °C; pH: 7.4 with HCl, 300 ± 5 mOsm/l with glucose; saturated with 95% O_2_ and 5% CO_2_). Using a vibratome (1000 Classic, Warner Instruments, Hamden, USA), sagittal brain slices of 300 µm thick were cut and placed in the same saline solution for 1 h at 34 °C. When recordings were done in slices, they were transferred to a submerged chamber and superfused at 5 ml/min with saline solution. When recordings were done in dissociated cells, the dorsal striatum was dissected from the slices and returned into the saline solution containing 10 mM HEPES plus 0.5 mg/ml of papain (*Carica papaya;* Calbiochem, Cat# 5125. San Diego CA) at 34 °C. After 20–25 min of digestion, the striatum slices were transferred to a low Ca^2+^ (0.4 mM CaCl_2_) saline solution. To obtain individual cells, the striatal slices were mechanical dissociated with a graded series of fire-polished Pasteur pipettes. The cell suspension (1 ml) was plated into a Petri dish mounted on the stage of an inverted microscope (Nikon Instruments, Melville, NY, 20 ×/0.4 NA). Cells were left for 10–15 min for neurons to adhere to the bottom of the dish. The dish contained 1 ml of the whole-cell recording saline solution (in mM): 0.001 tetrodotoxin (TTX), 140 NaCl, 3 KCl, 5 BaCl_2_, 2 MgCl_2_, 10 HEPES, and 10 glucose (pH: 7.4 with NaOH; 300 ± 5-mOsm/l with glucose). Thereafter, the cells were superfused at 1 ml/min with saline of the same composition at room temperature (approximate 25 °C). Tomato-positive neurons were visualized using a UV lamp (X-Cite; EXFO, Ontario, Canada; Fig. 1b). Dissociated neurons lack their distal dendrites and axon, so currents reported are somatic.

### Voltage clamp recordings of calcium currents

Voltage-clamp recordings were performed on identified striatal PV+ interneurons with 12–15 µM soma diameter and whole-cell capacitance of 6–7 pF with short or absent dendritic trunks [32, 34]. Patch pipettes of borosilicate glass (WPI, Sarasota, FL, USA) were pulled in a Flaming-Brown puller (Sutter Instrument Corporation, Novato, CA, USA) and fire polished prior to use. The internal saline solution contained (in mM): 180 N-methyl-d glucamine (NMDG), 40 HEPES, 10 EGTA, 4 MgCl_2_, 2 ATP, 0.4 GTP and 0.1 leupeptin (pH = 7.2 with H_2_SO_4_; 280 ± 5 mOsm/l; room temperature around 25 °C). Whole-cell recordings used electrodes with D.C. resistance of 3–6 MΩ in the bath. Liquid junction potentials (5-10 mV) were corrected. Recordings of Ca^2+^ currents were obtained with an Axopatch 200B patch-clamp amplifier (Axon Instruments, Foster City, CA, USA) and controlled and monitored with pClamp (version 8.2, RRID: rid_000085) and a 125 kHz DMA interface (Axon Instruments, Foster City, CA, USA). We recorded currents passing through Ca^2+^ channels using Ba^2+^ as a charge carrier as shown in previous articles [31, 34, 35]. Ba^2+^ is a potent K^+^ blocker. In addition, intracellular K^+^ was replaced by 180 mM NMDG. Na^+^ channels were blocked with 1 µM TTX. Currents isolated in this way were completely blocked by 200–400 µM Cd^2+^ (Fig. 1f) in this way, and for simplicity, we will refer to these currents as Ca^2+^ currents. Current–voltage relationships (I–V plots) were generated before and after drug application. Figure 1c shows representative Ca^2+^ currents evoked with 20 ms rectangular voltage commands from − 80 to 50 mV in 10 mV steps. Figure 1d shows a representative Ca^2+^ current in response to a voltage ramp command (0.7 mV/ms) from − 80 to 50 mV. When I–V plot from both methods coincide, space-clamp was considered acceptable (Fig. 1e). For clarity, most figures only show representative responses to voltage ramps.

### Current clamp recordings in slices

Current clamp recordings were performed with the patch clamp technique in the whole cell configuration of PV+ neurons of infected mice ranging in age 28–60 days. Sagittal slices (250–300 μm thick) were cut using a vibratome (1000 Classic, Warner Instruments, Hamden, USA), transferred to a recording chamber and superfused continuously with oxygenated saline solution (5 ml/min) at room temperature (~ 25 °C). Neurons within the striatum were visualized with infrared differential interference contrast videomicroscopy and PV+ neurons were identified using epifluorescent illumination with a 40 × immersion objective (0.8 NA; Nikon Instruments, Melville, NY). Micropipettes were pulled (Sutter Instrument, Novato, CA) from borosilicate glass tubes (WPI, Sarasota, FL) to an outer diameter of 1.5-mm for a final D.C. resistance of 4–6 MΩ when filled with internal saline. The internal solution contained (in mM): 120 KSO_3_CH_4_, 10 NaCl, 10 EGTA, 10 HEPES, 0.5 CaCl_2_, 2 MgCl_2_, 2 ATP-Mg, and 0.3 GTP-Na (pH = 7.3, 290 mOsM/l). Recordings were made with an Axopatch 200A amplifier (Axon Instruments, Foster City, CA) and data were acquired with the Im-Patch© software designed in the Lab View environment (freely available for download at im-patch.com). Evoked firing responses at different depolarizing membrane potentials were obtained before and after a selective dopamine receptor agonist was administered. Current–voltage relationships made in current-clamp mode superimposed tightly with those performed in voltage-clamp mode at steady state, suggesting that neither bridge balance, nor series resistance, represented a problem in our recordings.

Digitalized electrophysiological data were imported and analyzed into Origin v8, Microcal (Northampton, MA), and MatLab (The Mathworks Inc. Natick, MA). Data are presented as the mean ± standard error (SEM). Firing rate plots were made by taking firing rate at rheobase in the different pharmacological conditions (Fig. 5c). Free-distribution statistical tests Wilcoxon’s *T* test and Friedman, one-way ANOVA with post hoc Dunn’s tests were used to assess statistical significance between paired or unpaired samples comparisons. Statistical significance was defined by P-values below 0.05.

### Calcium imaging recordings

Calcium imaging recordings were obtained from PV+ neurons of mice infected with a Cre-dependent GCamp6f expression. Recordings were performed in saline solution containing (in mM): 126 NaCl, 2.5 KCl, 26 NaHCO_3_, 1.2 NaHPO_4_, 1 CaCl_2_, 1.3 MgCl_2_, 10 glucose, 0.2 thiourea and 0.2 of ascorbic acid (25 °C; pH: 7.4 with HCl, 300 ± 5 mOsm/l with glucose; saturated with 95% O_2_ and 5% CO_2_). For recordings, a microscope equipped with a 20 × 0.95 NA water-immersion objective (XLUMPlanFI, Olympus, Center Valley, PA) which has an image field of 750 × 750 μm, was used. To observe spontaneous changes in GCamp6f fluorescence intensity, light pulses at 488 nm (15–50 ms exposure) were delivered to the preparation with a Lambda LS illuminator (Sutter instruments, Petaluma, CA) connected to the microscope via optic fiber. Brief image sequences or movies (~ 180 s per epoch) were acquired with open access Im-Patch© software [6] at time intervals of 5–10 min during ≥ 60 min with a cooled digital camera (CoolSnap K4, Photometrics, Tucson, AZ) and 100–250 ms/image frame. Ca^2+^ entry was seen as spontaneous neuronal intrasomatic Ca^2+^ transients in PV+ neurons whose first time derivative reflects the time of electrical activity [36]. Activity of each cell was illustrated as dots in a raster plot.

### Inmunocytochemical procedures

PV-Cre mice were infected as described earlier. Mice were deeply anesthetized (see above) and perfused transcardially with a solution of 4% paraformaldehyde in PBS. Thereafter, animals were decapitated and their brains removed from the skull and fixed overnight with 4% paraformaldehyde in PBS. The brains were then cut on a vibratome into 40 μm slices that were incubated 30 min with 1% bovine albumina to block nonspecific binding sites and for 36 h with a rabbit polyclonal antibody against parvalbumin (anti PV 1:2000 Abcam dissolved in PBS containing 0.25% Triton-X). The slices were then rinsed thrice with PBS and incubated with a goat versus rabbit secondary antibody (1:200 Vector Laboratories, Burlingame, CA, dissolved in PBS containing 0.25% Triton-X) during 1 h. This antibody was conjugated with FITC (Vector Laboratories, Burlingame, CA). Samples were mounted with vectashield (Vector Laboratories, Burlingame, CA) and observed in a confocal microscope ZEISS LSM 700 (10 ×/1.0 NA) (n = 10).

### Drugs

For dissociated cell recordings, drugs were applied with a gravity-fed system that positioned a glass capillary tube 100 μm from the recording cell in the direction of superfusion flow. Solution changes were performed with a D.C. controlled microvalve system (Lee; Essex, CT, USA). This method allowed reversible drug applications [26, 33]. For current clamp recordings drugs were administered into the bath saline. Substances used were the DA receptor D1-like selective agonist SKF 81297 (Cat# S143), DA receptor D1-like antagonist SCH 23390 (Cat# 125941-87-9), Ca^2+^ Ca_V_1 antagonist nicardipine (Cat# N7510) all from Sigma-Aldrich-RBI (St Louis, MO, USA); Ca^2+^ Ca_V_2.2 blocker ω-conotoxin GVIA (Cat# C-300), Ca^2+^ Ca_V_3 blocker TTA-P2 (Cat# T-155), Ca^2+^ Ca_V_2.3 blocker SNX-482 (Cat# RTS-500), Na^+^ blocker tetrodotoxin (TTX) (Cat# T-550) from Alomone Laboratories (Israel) and Ca^2+^ Ca_V_2.1 blocker ω-agatoxin TK (Cat# 4294-s) from Peptides International (Louisville, KY).

### Data analysis

Collected digitalized data were analyzed and plotted using commercial software (Origin v8, Microcal, Northampton, MA, USA; RIDD: rid_000069). We report mean ± SEM of peak Ca^2+^ currents changes for dissociated FSI recordings without assuming normal distributions. We also used the 5, 25, 50 (median), 75 and 95 percentile ranges of absolute current values represented as Tukey box plots. Friedman, Kruskal–Wallis or Wilcoxon test with post hoc Dunn for multiple comparisons tests were used (signaled in each Result). Friedman and Wilcoxon test were used when we compared the same samples in two or three different conditions (before, during and after application of a drug). P< 0.05 was used as significance threshold. Analysis was conducted by GraphPad Prism 6.01 (La Joya, CA). Here, Ba^2+^ currents are reported as Ca^2+^ currents and graphs summarizing sampling results are illustrated. For current clamp recordings, we report mean ± SEM of firing rate. For calcium imaging experiments, activity of each FSI was determined as the total number of active frames/total number of frames. Finally, to quantify the amount of activity on each experiment, a cumulative activity plot was built on each condition.

### Contribution of each class of Ca^2+^ channel to the whole-cell Ca^2+^ current

The method to obtain the average contribution of a given class of Ca^2+^ channel to the whole cell Ca^2+^ current was described in previous work [37]. Briefly, to approximate the contribution of each class of Ca^2+^ channel, the amount of Ca^2+^ current blocked by a given antagonist: nicardipine, ω-conotoxin GVIA (ω-CgTx), ω-agatoxin TK (ω-AgTx), TTA-P2 (TTA) and SNX-482 (SNX) was obtained by subtraction in the same or different experiments. Hardly all antagonists could be tested in a single experiment, but the amount blocked by each antagonist was taken no matter the number or order of the antagonists tested. This amount of blocked current was defined as the contribution of that specific channel class to the whole-cell control Ca^2+^ current normalized to 100% without any antagonist. Thereafter the data was introduced in the following system of linear equations:

$$\begin{array}{*{20}l} {0_{{{\text{X}}1}} + N_{{{\text{X}}2}} + PQ_{{{\text{X}}3}} + T_{{{\text{X}}4}} + R_{{{\text{X}}5}} = A} \hfill \\ {L_{{{\text{X}}1}} + 0_{{{\text{X}}2}} + PQ_{{{\text{X}}3}} + T_{{{\text{X}}4}} + R_{{{\text{X}}5}} = B} \hfill \\ {L_{{{\text{X}}1}} + N_{{{\text{X}}2}} + 0_{{{\text{X}}3}} + T_{{{\text{X}}4}} + R_{{{\text{X}}5}} = C} \hfill \\ {L_{{{\text{X}}1}} + N_{{{\text{X}}2}} + PQ_{{{\text{X}}3}} + 0_{{{\text{X}}4}} + R_{{{\text{X}}5}} = D} \hfill \\ {L_{{{\text{X}}1}} + N_{{{\text{X}}2}} + PQ_{{{\text{X}}3}} + T_{{{\text{X}}4}} + 0_{{{\text{X}}5}} = E} \hfill \\ \end{array}$$

where *L, PQ, N, T* and *R* are the contributions in percentage (± SEM) of each channel class: Ca_V_1, Ca_V_2.2, Ca_V_2.1, Ca_V_3 and Ca_V_2.3 to the whole-cell Ca^2+^ current. For example, PQ refers to the current blockade by the selective P/Q type Ca^2+^ channel antagonist (ω-AgTx). Zero in the linear equation system means a blockade of a given Ca^2+^ channel class, thus, coefficients L, N, PQ, T or R were replaced by zero when the corresponding Ca^2+^ channel class was blocked. *A, B, C, D* or *E* stand for the mean percentage of Ca^2+^ current in the control (100%) with one channel class blocked (< 100%). Subscripts *X1*–*X5* are the unknown variables, in other words, the values that multiply the coefficients *L, N, PQ, T* and *R* in order to determine percentage contribution of each channel to the whole-cell Ca^2+^ current.

## Results

Striatal FSI express the Ca^2+^ binding protein PV [2, 38] and activation of the D5-type DA receptor from the D1-like class depolarizes FSI neurons to increase their action potentials (APs) firing rate [20, 21]. However, a final effector and whether DA enhances or decreases Ca^2+^ currents in FSI is unknown. With the help of PV-Cre transgenic mice we explored whether DA receptor actions in acutely dissociated striatal FSI modulate Ca^2+^ currents. In our experiments Na^+^ and K^+^ channels were blocked (Fig. 1c, d).

### Ca^2+^ channels expressed in striatal FSI

FSI were identified using mice expressing Cre-recombinase under the control of the PV promoter (PV-Cre) and stereotaxic injections of an adeno-associated virus into the dorsal striatum allowed expression of a fluorescent protein (td-Tomato) only in striatal FSI. Acutely dissociated neurons were used in the first part of this study to avoid any indirect inputs from afferents, gap junctions, dendritic or axonal inputs. First, we estimated which Ca^2+^ channels are present in the soma and nearby dendrites of FSI and their percentage contribution to the overall whole cell Ca^2+^ current optimizing space clamp (Fig. 1e and current isolation; see Methods). Ca^2+^ entry through different calcium channels exert different and complex responses that vary in different cell types and localization within the cell body [37 for a review], therefore, one goal of this study was to determine the classes of voltage gated Ca^2+^ channels present in striatal FSI. Representative experiments with time courses of Ca^2+^ current amplitudes before and after application of specific channels antagonists is shown in Fig. 2a, b and percentage contribution of each Ca^2+^ channel class is summarized in Table 1. Antagonists were administered in different order or alone, and the current they reduced was compared with the whole cell Ca^2+^ current without any antagonist (see Methods). To determine whether Ca_V_1 (L-type) contributed to the whole-cell current, application of nicardipine, a specific Ca_V_1 Ca^2+^ channels antagonist was examined. As shown in Fig. 2a, nicardipine at saturating concentrations (20 µM) reduces whole-cell current amplitude by 38 ± 1.1%. This reduction was significant when whole cell current was normalized to 100% for the current without any antagonist in control conditions (n = 12; P = 0.0001; Kruskal–Wallis ANOVA with post hoc Dunn’s test, used in this and next antagonists cases, percentages were obtained with the system of linear equations described in the Methods and compared to whole cell Ca^2+^ current without any drugs). This large amount of current flux through Ca_V_1 Ca^2+^ channel might contribute to neuronal depolarization and AP generation after addition of D1-like agonist, since this current have a slow voltage-dependent inactivation [20, 25, 39, 40]; a main hypothesis tested below.

**Table 1:** Contribution in percentage of the whole-cell Ca^2+^ current for each class of Ca^2+^ channel

Antagonist	Nicardipine	ω-conotoxin GVIA	ω-Agatoxin TK	SNX-482	TTA-P2
Concentration	20 µM	1 µM	1 µM	1 µM	1 µM
Ca^2+^ channel antagonist	Ca_V_1	Ca_V_2.2	Ca_V_2.1	Ca_V_2.3	Ca_V_3
% of current blocked (mean ± S.E.M)	38 ± 1.1	23.4 ± 0.7	11.1 ± 1.4	20 ± 2	7.4 ± 2.3
n	12	14	14	6	13
p	0.0001	0.0001	0.0033	0.0009	0.0202

The first row (not counting the title) indicates the Ca^2+^ channel antagonist used. The second row contains saturating concentrations used. Third row stands for the specific Ca^2+^ channel class that was blocked. The fourth row displays the mean ± SEM of Ca^2+^ current blocked in percentage by each antagonist. Antagonists were tested alone or in sequence in different orders. Percentages were obtained from a system of linear equations that used data from all experiments (see Materials and methods). The fifth row shows samples size: the number of neurons tested with each antagonist. The sixth row indicates statistical significance or P-value of percentage blockade by each antagonist as compared to whole-cell current average before adding any antagonist (Kruskal–Wallis ANOVA with post hoc Dunn’s test of each paired comparison against the control: whole-cell Ca^2+^ current)

Ca_V_2.2 (N) contribute to 23.4 ± 0.7% as revealed by 1 µM of ω-conotoxin GVIA (ω-CgTx) blockade, a specific Ca_V_2.2 channel antagonist (Fig. 2a, Table 1; P = 0.0001; n = 14). Contribution of Ca_V_2.1 (P/Q) was 11.1 ± 1.4% disclosed by ω-agatoxin TK (1 µM; Fig. 2a; Table 1; P = 0.0033). 1 µM SNX-482 revealed the presence of Ca_V_2.3 (R) Ca^2+^ channels: 20 ± 2% (Fig. 2b; Table 1; n = 6; P = 0.0009). Finally, Ca_V_3 (T) Ca^2+^ channels contribute with 7.4 ± 2.3% (Fig. 2b; Table 1; n = 13; P = 0.0202). To conclude, representative components of high voltage gated (HVA) and low voltage gated (LVA) Ca^2+^ channels are present in FSI. The specific type and role of each of these channels is a matter of future studies out of the scope of the present work. We next concentrate on Ca_V_1 Ca^2+^ channels which provide much of the whole cell Ca^2+^ current.

### Activation of D1-class receptor enhances Ca^2+^ currents in acutely dissociated FSI

To know whether DA has effect on FSI Ca^2+^ currents, we performed whole-cell recordings in dissociated and identified FSI cells. Time course of peak current is shown in I–V plots of Fig. 3a before, during and after the administration of 10 µM of the DA receptor D1-like agonist SKF-81297 (SKF). SKF enhanced whole-cell Ca^2+^ currents in all FSI tested by an average of 34 ± 14% (Fig. 3a, b; *n* = 8. Friedman ANOVA test F_2,14_ = 13, P = 0.0003; *P < 0.05, **P < 0.01; with post hoc Dunn’s test). Note that Ca^2+^ current returns to values similar to the control when the agonist is washed-off. Representative I–V plots (Fig. 3a right) are shown at different moments of the time course. Box plot in Fig. 3b summarizes the results from the previous sample of experiments showing that SKF actions were significant. The effect of the SKF was blocked by the presence of 100 nM of the DA receptor D1-like antagonist SCH 23390 (SCH; Fig. 3c). Removal of SCH leads to an increase in Ca^2+^ current in the presence of SKF showing that activation of D1-like DA receptors, most probably D5 [20, 21], enhances Ca^2+^ currents in FSI. No significant differences were found comparing controls and the combination SCH/SKF (Fig. 3d) (n = 8; Wilcoxon T test P > 0.9999). These results are consistent with the expression of D1-class DA receptors in FSI and show that DA enhances Ca^2+^ currents through the activation of these receptors.

### D1-like receptors modulate Ca_V_1 Ca^2+^ channel current

To further investigate the role of DA on Ca^2+^ currents we performed experiments blocking Ca^2+^ currents while activating DA receptors. Because Ca_V_1 Ca^2+^ channels provide the most to the whole cell Ca^2+^ current we first blocked them [25]. Time course of peak current amplitude (top) and a representative I–V plots (bottom) are illustrated in Fig. 4a showing first, the action of 20 µM of the selective antagonist nicardipine on Ca_V_1 currents. Then the action of subsequent application of the D1-like agonist SKF is shown. Nicardipine reduced Ca^2+^ current by 55.4 ± 7.8% in this sample (Fig. 4a, b; n = 8; Friedman ANOVA test F_2,14_ = 13, P = 0.0003; *P < 0.05, **P < 0.01 with post hoc Dunn’s test). Note that in the presence of nicardipine subsequent addition of 10 µM SKF fail to elicit any change in the remaining Ca^2+^ current (P = 0.9999; Dunn’s test). We conclude that the action of nicardipine occluded the action of SKF and therefore Ca_V_1 Ca^2+^ channels are the final effectors of DA receptor modulation; without excluding other classes of channels (Na^+^, K^+^). This action is similar to that found in striatonigral projection neurons expressing D1-like receptors in both cell bodies and terminals [15, 41, 42]. It is also inferred that with respect to Ca^2+^ currents, there is no other effector for D1- receptor modulation in FSI.

### D1-like receptors enhance FSI firing rate by modulating Ca_V_1 Ca^2+^ channels

We then asked whether Ca_V_1 Ca^2+^ channels modulation is robust enough to explain, in part, the increase in firing rate due to D1-like receptor modulation in FSI [20, 21]. Whole-cell current-clamp experiments on identified PV+ neurons in slices of transfected PV-Cre mice were performed. FSI in the dorsal striatum were identified based on adeno-associated virus containing td-Tomato (Fig. 5a top left; see Methods) and corroborated with an antibody against PV conjugated to fluorescein isothiocyanate (FITC; Fig. 5a middle left). Merge is at the bottom in Fig. 5a. FSI were also identified by their electrophysiological phenotype: ability to fire at high firing rates with almost no frequency adaptation as well as stuttering (Fig. 5b). Representative recordings evoked by different intracellular current injections are shown in Fig. 5b: 10 µM SKF induced increases in mean firing rate at rheobase (n = 6; P < 0.0001; Friedman ANOVA with post hoc Dunn’s test using average firing rate after 300 pA. Fig. 5c). Notably, subsequent administration of the Ca_V_1 antagonist, nicardipine (20 µM), reversed in part the increase in firing rate induced by SKF. The same was true for a D1-receptor antagonist SCH (Fig. 5d; n = 4). Several seconds had to be taken between stimuli that evoke firing, before and after drugs administration, since in our hands, intensity-frequency plots exhibited hysteresis (adverse effect), a phenomenon that needs further investigation but out of the scope of the present report.

### Activation of D1-class receptors enhances FSI activity in the dorsal striatal microcircuit

Finally, we asked whether activation of DA D1-class receptors can enhance the number of active FSI within the striatal microcircuit by enhancing Ca_V_1 Ca^2+^ current. To test this hypothesis we performed calcium imaging experiments with single cell resolution [36] in slices from PV-Cre transgenic mice expressing GCaMP6f as a fluorophore. Using this technique we recorded spontaneous calcium transients of several PV+ neurons in different slices from three mice. The time derivative of these calcium transients indicates their firing time [36]. FSI may fire spontaneously in control conditions together with the firing of other striatal neurons. To avoid confounds we only graphed FSI activity using raster plots where dots in each row represent the moments of activity of single neurons (Methods). The firing of FSI from different slices (n = 6) are plotted together (Fig. 6a) as previously described [6, 36, 43]. Changes in fluorescence were obtained before, during and after application of SKF and SKF plus nicardipine. Raster plot of active FSI during a period of 13 min recording is shown in Fig. 6a (n = 33 identified FSI). The left panel in Fig. 6a shows the basal FSI activity in the striatal microcircuit without adding any excitatory drive or drug. Notice scarce FSI activity in control conditions. In contrast, administration of 10 µM SKF to the bath saline increased the number of active FSI (Fig. 6a middle panel). The subsequent addition of nicardipine in the presence of SKF reduced, but not completely reversed the enhanced activity. Figure 6b shows cumulative activity of all FSI neurons along time [43] and Fig. 6c shows activity probability under each condition (mean ± SEM). To compare activity over time a cellular activity value for each neuron at each condition was calculated (frames with active neuron/total number of frames). In control conditions cellular activity was 0.03 ± 0.008, SKF raised activity to 0.07 ± 0.008 (Fig. 6b, c; Friedman ANOVA F_2, 64_ = 29.63, P < 0.0001; post hoc Dunn’s test). This result indicates that DA increases the number of FSI firing within the striatal microcircuit in agreement with data from dissociated neurons and slice experiments. 20 µM nicardipine (Fig. 6a right panel) reduced FSI activity to 0.05 ± 0.008 (Fig. 6b, c; P < 0.001, Dunn’s test).

## Discussion

A summary of original data and findings of the present work follow: (1) all major classes of voltage gated Ca^2+^ channels are present in striatal FSI (Ca_V_1, C_V_2.1-3; Ca_V_3). These results were obtained in voltage-clamp mode in identified dissociated FSI. Specific channel subtypes are still in need of investigation. (2) Contributions in percentage of each Ca^2+^ channel class are reported. Ca_V_1 channels represent much of the whole cell Ca^2+^ current. (3) DA D_1_-class receptors, probably D_5_-type [21], up-modulate Ca_V_1 carried current. (4) The Ca_V_1 class is the only Ca^2+^ channel modulated by DA in FSI. This modulation is occluded by a previous administration of nicardipine or blocked by the antagonist SCH [20]. (5) Modulation of Ca_V_1 Ca^2+^ channels is reflected in an increase in firing rate of FSI. These data were obtained in slices in current clamp mode. (6) Ca^2+^-imaging recordings of several identified FSI obtained from different slices/animals showed that DA increases the number of FSI that are active in the striatal microcircuit. To our knowledge these are the first evidences of a molecular final effector for the DA-dependent modulation in striatal FSI, leading to a better understanding of the DA actions in the striatum.

### Ca^2+^ channels expressed in FSI

Using pharmacological tools here we demonstrate that identified FSI from transgenic animals may be isolated in enough number to study the ion channels they express with whole-cell voltage clamp techniques in acutely dissociated preparations, thus eliminating indirect sources or confounds such as inputs from other neurons as well as gap junctions and chemical synapses between FSI themselves, this maneuver allows study specific Ca^2+^ currents [30, 33, 34, 37]. Striatal FSI seem to express all classes of voltage gated Ca^2+^ channels, HVA (Ca_V_1, Ca_V_2.1, Ca_V_2.2 and Ca_V_2.3) and LVA (Ca_V_3), although, their contributions vary (Fig. 2a, b and Table 1). On average, Ca_V_1 channels contribute the most to the whole cell Ca^2+^ current followed by Ca_V_2.2 and Ca_V_2.3 channels that altogether make up to more than 80% of the whole cell Ca^2+^ current. Ca_V_2.1 and Ca_V_3 make up the remaining current. Together with other ion channels [44–46], the studied Ca^2+^ channels shape the characteristic firing properties [28, 44–46] of FSI and may be orchestrated by signaling pathways as it occurs in striatal projection neurons (SPNs) [27, 30, 37, 40, 41]. Although it was not the goal of the present study to explore the role of each Ca^2+^ channel encountered, the variety found may imply that each channel has a specific role and ways to be modulated [6, 13, 14, 30, 35, 37, 40, 41]. Pathologies associated with striatal FSI, such as anxiety-like behaviors, schizophrenia or disorders such as Tourette’s and Huntington’s disease as well as some channelopathies [7, 47–51] may use this preparation to study associated changes.

### Dopaminergic modulation of striatal FSI Ca^2+^ currents

A selective D1-class DA receptor agonist, SKF-81297, was used to investigate dopaminergic modulation. The DA receptor agonist enhanced Ca^2+^ currents specifically carried by Ca_V_1 channels in FSI. A previous blockade of these channels with a dihydropyridine, nicardipine, completely occluded the action of the DA receptor agonist. Notably, this is similar to the dopaminergic modulation found in direct basal ganglia projection neurons (dSPNs) except that in the present case there was no need to block intracellular phosphatases [20].

Current through Ca_V_1 channels has been associated with enhanced evoked depolarization and discharge facilitation [41, 52, 53]. Current clamp experiments in slices showed that this is also the case for striatal FSI as well as other neurons [37]. Ca_V_1 also induces short-term synaptic depression and facilitates GABA release in SNr [53, 54]. Blockade of enhanced firing by nicardipine shows that Ca_V_1 channels are in part responsible for these functions in striatal FSI. It would be interesting to know if FSI from other nuclei express this modulation or if it is particular for striatal FSI.

In addition, dynamic Ca^2+^ imaging of identified FSI with single cell resolution showed that the firing of these interneurons is enhanced in the striatal microcircuit by dopaminergic modulation. This action was partially blocked by nicardipine, lasted for several minutes without overt desensitization, suggesting that D1-class receptor activation, probably D5-type, increases feed-forward inhibition in the microcircuit [1, 55, 56]. Network analyses of this action in control and disease in vitro models [6] deserve further study. In addition, calcium recording was not performed in vivo, so the impact of excitatory drive from cortex and thalamus were not evaluated in the DAergic actions reported, although, in vitro studies have shown similar suprathreshold responses on thalamic and cortical stimuli, suggesting that both sources produces similar feed-forward inhibition on SPN [11]. Given that the resonant frequency of FSI is within the gamma band [57], it may be logical to infer that DA modulation favors gamma (Piper) rhythms within neuronal circuits [4, 58–60]. On the other hand, aberrant or excessive gamma rhythms may be present during schizophrenia and L-DOPA induced dyskinesia [8, 18, 61].

However, the number of dopamine activated FSI within the striatal microcircuit does not return to control conditions after Ca_V_1 Ca^2+^ channels are blocked. There could be various reasons for this behavior. One is that the DA receptor agonist not only affects FSI within the circuit, but turns on network activity in a way that does not return to control even after blocking Ca_V_1 in FSI [19, 62]. Another explanation is that circuit activity or DA activates other ion channels in FSI [19]. Finally, FSI form networks of interconnected neurons both electrically and chemically [1, 2, 11]. This last property may correlate FSI firing making hard to study their individual cell responses in striatal brain slices.

## Conclusion

To our knowledge this is the first demonstration that Ca_V_1 channels are final effectors of DA modulation in FSI. In addition, we show the classes of Ca^2+^ channels that striatal FSI express and show evidence that Ca_V_1 are the only ones modulated by D1-class receptors activation. Enhancement of Ca_V_1 channels is a main cause for the increase in excitability of these interneurons due to DA receptors signaling, and collectively, this modulation increases the number of active FSI during striatal microcircuit operation. The demonstration that identified interneurons can be isolated for recording opens the pathway for future studies such as: to study other current classes (K^+^, Na^+^) and their modulation in interneurons in space clamp conditions, it also suggests comparisons between current phenotypes between FSIs from the striatum and other nucleus such as the cortex, and finally, quantitative single cell PCR may be used to prove whether different regions of the striatum possess different types of FSIs.

## Abbreviations

FSI: fast spiking interneurons
PV: parvalbumin
HVA: high voltage activated
LVA: low voltage activated
SPN: spiny projection neurons
dSPN: striatonigral projection neurons
TTX: tetrodotoxin
SNc: sustantia nigra pars compacta
ChAT: giant cholinergic interneurons
DA: dopamine
HEPES: 4-(2-hydroxyethyl)-1-piperazineethanesulfonic acid
NMDG: N-methyl-d glucamine
eGFP: enhanced green fluorescent protein
iFr: instantaneous firing rate
